# Feasibility and Preliminary Efficacy of α–Calcium Sulfate Hemihydrate in Socket Preservation: Protocol for a Pilot Randomized Controlled Trial

**DOI:** 10.2196/49922

**Published:** 2024-07-19

**Authors:** Muhammad Ruslin, Nurlindah Hamrun, Andi Tajrin, Andi Sitti Hajrah Yusuf, Rifaat Nurrahma, Diandra Sabrina Natsir-Kalla, Yossy Yoanita Ariestiana, Mukhtar Nur Anam, Chung-Ming Liu, Keng-Liang Ou

**Affiliations:** 1 Department of Oral and Maxillofacial Surgery Faculty of Dentistry Hasanuddin University Makassar Indonesia; 2 Department of Oral Biology Faculty of Dentistry Hasanuddin University Makassar Indonesia; 3 Hasanuddin University Dental Hospital Makassar Indonesia; 4 Department of Prosthodontic Faculty of Dentistry Hasanuddin University Makassar Indonesia; 5 Department of Oral and Maxillofacial Surgery/Oral Pathology, Amsterdam University Medical Centers and Academic Centre for Dentistry Amsterdam Vrije Universiteit Amsterdam Amsterdam Movement Sciences Amsterdam Netherlands; 6 Department of Biochemistry Faculty of Medicine Hasanuddin University Makassar Indonesia; 7 Department of Biomedical Engineering College of Biomedical Engineering China Medical University Taichung Taiwan; 8 Graduate Institute of Dental Science College of Dentistry China Medical University Taichung Taiwan; 9 Department of Dentistry Taipei Medical University-Shuang Ho Hospital New Taipei City Taiwan; 10 3D Global Biotech Inc (Spin-Off Company from Taipei Medical University) New Taipei City Taiwan; 11 Department of Oral Hygiene Care Ching Kuo Institute of Management and Health Keelung Taiwan; 12 Division of Clinical Cariology and Endodontology, Department of Oral Rehabilitation School of Dentistry Health Sciences University of Hokkaido Hokkaido Japan

**Keywords:** α–calcium sulfate hemihydrate, α-CSH, bone graft, bone regeneration, socket preservation, pilot, feasibility, efficacy, impacted tooth, tooth extraction, radiographic imaging, bone, dentist, dentistry, dental, bone resorption, graft, bone loss, alveolar bone, regeneration

## Abstract

**Background:**

Tooth extraction procedures often lead to bone resorption, which can have adverse effects on the dimensions of the alveolar ridge. Research has shown that socket preservation techniques using bone graft substitutes can effectively minimize early bone loss in such cases. α–calcium sulfate hemihydrate (α-CSH) has garnered significant attention as a potential bone graft material due to its favorable properties, including osteoconductivity, angiogenic potential, and biocompatibility. Considering these facts, we developed a preliminary protocol for applying α-CSH in addressing alveolar bone loss following tooth extraction.

**Objective:**

This research’s general objective is to evaluate the feasibility and initial effectiveness of α-CSH as bone-inducing graft material for socket preservation after tooth extraction.

**Methods:**

This preliminary clinical trial will involve 30 fresh extraction sockets from individuals aged 18-35 years. The participants will be divided into 2 groups: one group will receive α-CSH graft material after tooth extraction for socket preservation, while the other group will not receive any graft material. Throughout the study, the participants will be closely monitored for safety measures, which will include clinical examinations, radiographic imaging, and blood tests. Radiographic imaging will be used extensively to assist the progress of bone formation.

**Results:**

The study commenced enrollment in August 2022 and is scheduled to conclude post assessments and analyses by the end of 2023. The results of the study are anticipated to be accessible in late 2024.

**Conclusions:**

This clinical study represents the initial investigation in humans to assess the feasibility and efficacy of α-CSH in alveolar bone regeneration. We hypothesize that the inclusion of α-CSH can greatly expedite the process of bone formation within fresh sockets, resulting in a swift restoration of bone height without the disadvantages associated with harvesting autogenous bone graft.

**Trial Registration:**

Indonesia Registry Center INA-D02FAHP; https://tinyurl.com/2jnf6n3s

**International Registered Report Identifier (IRRID):**

DERR1-10.2196/49922

## Introduction

Bone resorption is an ongoing and irreversible process that leads to changes in both hard and soft tissue dimensions, particularly occurring immediately after tooth extraction [1,2]. Various factors, such as chronic periodontitis, alveolar cleft, prolonged edentulous state, and tooth morphology, can contribute to alveolar bone resorption [3,4]. In addition, anatomical, prosthetic, functional, and metabolic factors have been identified as influencing factors in alveolar ridge resorption [5,6]. A previous study reported an average of 50% vertical resorption and 20% horizontal bone resorption occurring 8 weeks after tooth extraction [7]. Considering these findings, the dimensional changes in the alveolar bone following tooth loss raise significant concerns in oral rehabilitation [6,7].

In recent years, there has been a growing emphasis on developing techniques to preserve alveolar bone volume following tooth extraction [2,6,8]. Socket preservation procedures are recommended when tooth extraction significantly impacts the height of the buccal bone [9,10]. These techniques aim to minimize the biological resorption of the buccal bone, which facilitates implant placement and reduces the need for future bone augmentation procedures. Studies have shown that socket preservation techniques lead to greater bone volume compared to procedures without socket preservation [8,11]. In the future, sites that undergo grafting after tooth extraction are likely to require fewer bone augmentation treatments, particularly if implantation is planned [1,10]. Various biomaterials have been used to graft fresh extraction sockets, and most have demonstrated positive clinical outcomes [8,12,13].

α–calcium sulfate hemihydrate (α-CSH) has gained significant recognition as a bone graft substitute material with outstanding biocompatibility [14,15]. It has successfully repaired various bone defects, including resorbed maxilla, postextraction socket preservation, and significant segmental bone defects [14-16]. The α-CSH is regarded as a leading bone replacement material due to its ability to promote bone ingrowth and its resemblance to autologous bone [14,15,17]. Notably, it exhibits excellent workability, high self-setting strength, impressive osteoconduction properties, and the capacity to induce hemostasis and angiogenesis [14,17,18]. Despite these remarkable biological characteristics, this material still has to function better in clinical settings [15]. This study aims to evaluate the feasibility and preliminary efficacy of α-CSH as a bone-inducing graft material in socket preservation procedures following tooth extraction. Our hypothesis suggests that α-CSH will serve as a safe, efficient, and effective alternative to conventional autografts, as its regenerative properties complement its osteoconductive nature.

## Methods

### Ethical Considerations

The clinical trial was approved by the Ethics and Research Committee of Faculty of Medicine, Hasanuddin University, Makassar, Indonesia, with code number 504/UN4.6.4.5.31/PP36/2022. The study was designed according to the principles of the Declaration of Helsinki and will conform to the CONSORT (Consolidated Standards on Reporting Trials) statement [19].

Before participating in the study, all individuals must provide written informed consent, signifying their voluntary agreement to participate. Participants will have the opportunity to seek clarification on any aspects of the study and review all procedures, data collection methods, and data analysis protocols before signing the consent form. The informed consent document will comprehensively outline the research’s purpose, procedures, potential risks and benefits, as well as the confidentiality and security measures pertaining to the collected data. Participants will be explicitly informed of their entitlement to withdraw from the study at any point without experiencing any adverse consequences on their treatment.

Strict privacy and security protocols were implemented, and all study data were stored in a password-protected secure database. Each participant will receive a unique study code, and data sharing with operators and research assessors will be done in an encrypted format that maintains patient privacy. Participants will not be provided with any form of remuneration for their involvement in the study. Nonetheless, they will receive complimentary examination and treatment related to the trial without incurring any costs.

### Recruitment

**Figure 1:**
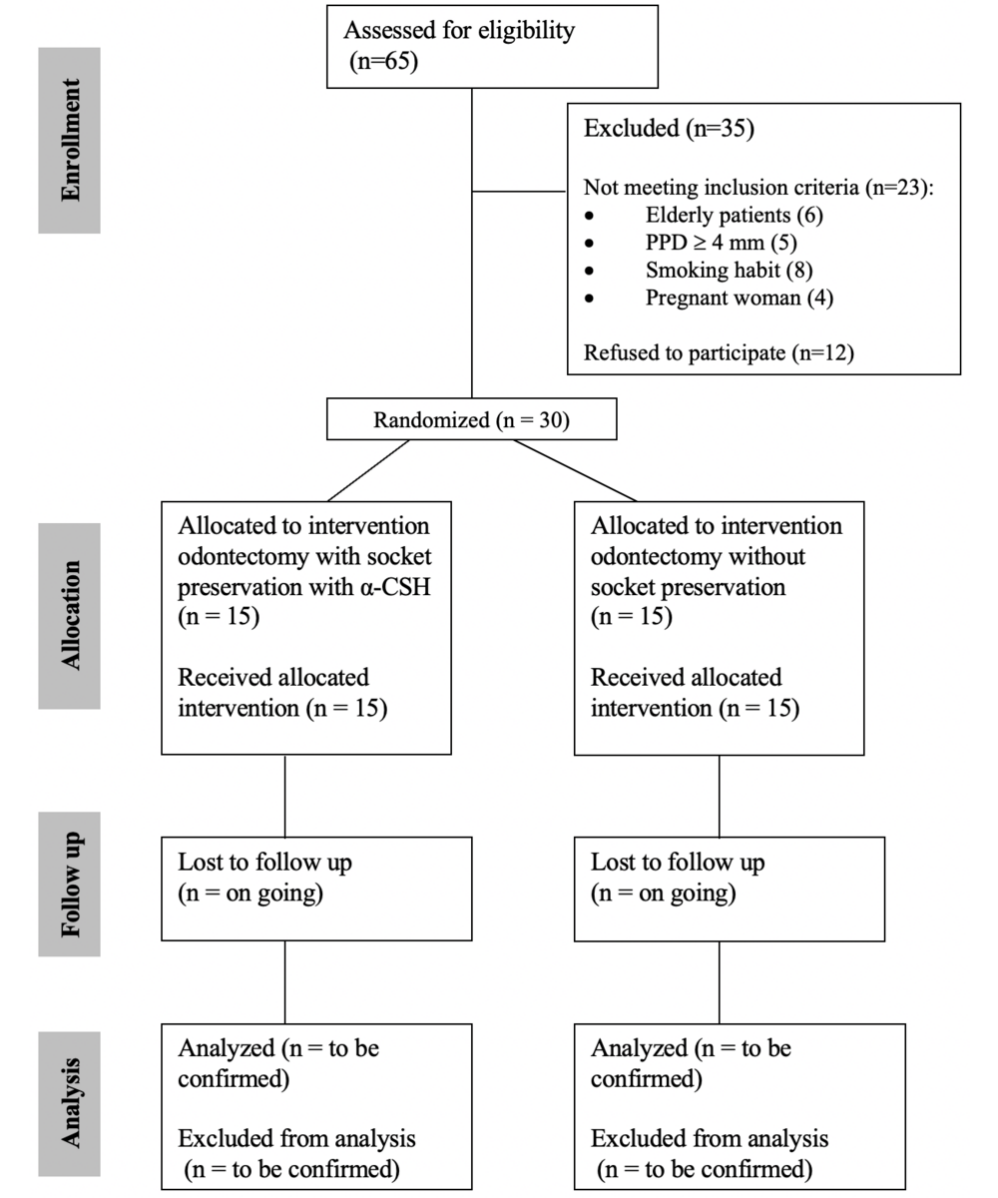
CONSORT (Consolidated Standards of Reporting Trials) flowchart diagram. α-CSH: α–calcium sulfate hemihydrate; PPD: probing pocket depth.

Figure 1 presents a simplified diagram that outlines the study protocol based on the CONSORT diagram. The recruitment of participants and the implementation of the trial will take place at Hasanuddin Dental Hospital, Makassar, Indonesia. This hospital is the primary institution in the designated region that handles the majority of oral surgery cases. To identify eligible participants, all individuals presenting to the Department of Oral and Maxillofacial Surgery with a chief complaint related to the mandibular third molar will undergo screening conducted by the study investigators.

Before enrollment, all participants will be provided with detailed information regarding the risks and potential complications associated with the procedure, such as bleeding, infection, cheek asymmetry, parotid duct injury, potential injury to facial nerve branches, and the unlikely event of nonclosure. Informed consent will be obtained from each participant after ensuring their understanding and agreement.

### Study Design

This prospective control clinical trial will be conducted at a single center and will involve 30 targeted extraction sockets after the removal of impacted mandibular third molars. This case is chosen as it represents a common scenario requiring extraction and often involving the removal of a significant amount of bone, making it an ideal model for studying human alveolar bone loss for socket preservation. The study aims to evaluate the efficacy of a high-purity α-CSH bioceramic (CaSO_4_·0.5H_2_O), synthesized from calcium sulfate dihydrate (CSD) without the use of chemical reagents, as a bone graft material in the alveolar bone loss case.

The 30 extraction sockets will be randomly assigned into 2 groups: 15 will receive α-CSH as a bone graft, while the other 15 will not receive any graft material. The main end point will be evaluated 6 months into the procedure. During follow-up visits, any adverse events or serious adverse events will be carefully documented, and clinical assessments will be conducted as specified in the “intervention section.” All patients will undergo regular monitoring through clinical examinations, radiographs, lab tests, and any additional necessary procedures. Ultimately, the study will publish a comprehensive report on the feasibility and potential efficacy of the α-CSH approach in promoting bone formation, regardless of the outcome.

### Inclusion and Exclusion Criteria

**Figure 2:**
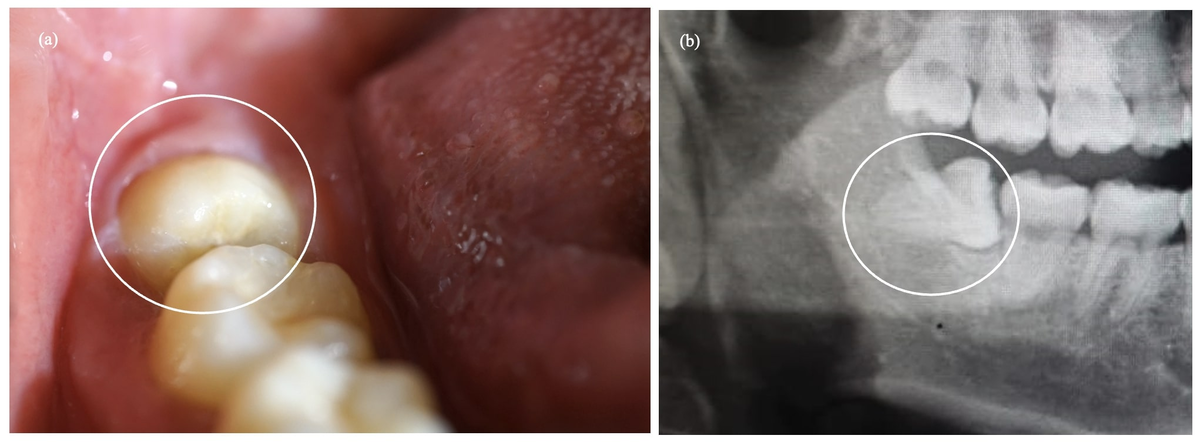
Preoperative image of the clinical and radiographic condition of the patients.

To be eligible for participation in this clinical trial, patients must meet specific inclusion criteria, which are as follows: being a healthy male or female; aged 18-35 years old; providing written informed consent to participate in the trial; having an average blood count within normal range; presenting with impacted mandibular third molars that require extraction. The diagnostic indications for tooth extractions include caries, orthodontic, and preventive measures. In particular, based on the relation of the impacted mandibular third molar to the ramus, the impacted teeth will be restricted to Class I and Class II as per the Pell and Gregory classification [20,21]. Patients had to demonstrate good oral hygiene, maintain healthy periodontium, and have a clinical attachment loss score of ≤2 mm and a probing pocket depth (PPD) of <4 mm. Clinical and radiographic images taken before the operation are depicted in Figure 2.

Patients will be disqualified from participating in the study if they meet any of the following exclusion criteria: impacted teeth concerning their position relative to the occlusal plane fall within the Pell and Gregory classification's level C category [20,21]; presence of uncontrolled or untreated periodontal disease; existence of acute abscesses or active infections that occur close to the targeted surgical site; presence of systemic diseases or systemic/local infections; recent receipt of chemotherapy, radiotherapy, immunosuppressant medications, or anticoagulants that could potentially interfere with the healing process; previous administration of bone growth–inducing substances, malnutrition, or current active influenza; regular smoking habit of more than 10 cigarettes per day; being pregnant, breastfeeding, or participating in a fertilization program.

### Withdrawal of Participants

Participants have the right to withdraw from the study at any time and for any reason without facing any negative consequences. In cases where a participant has urgent medical needs, the researcher may choose to exclude them from further participation in the study. Participants who choose to discontinue the study before completing all surgical procedures and follow-up visits will be considered dropouts.

### Material Preparations

The high-purity α-CSH bioceramic was synthesized from calcium sulfate dihydrate (Acros Organics, Morris Plains, NJ) using a microwave irradiation technique, following the methodology outlined in our previous study [14,17]. In brief, 2 g of CSD powder were mixed with 10 mL of deionized water. Following this procedure, the resulting mixture, along with a magnetic stirring bar, was placed in a 55-ml Teflon pressure tube at approximately 350 pounds per square inch (psi). The mixture was then subjected to microwave irradiation at 800 watts and 160 °C for 10 minutes. After synthesis, the sample was filtered, washed with ethanol, then dried in an oven at 60 °C for 8 hours, and finally ground into a powder.

### Interventions

Prior to the surgical procedures, a preoperative panoramic x-ray and cone-beam computed tomography (CBCT) scan will be conducted to evaluate the tooth position and the surrounding bone structure. A complete blood count (CBC) will also be performed to ensure the safety of all patients undergoing surgical procedures and receiving bone grafts. Furthermore, the PPD score will be measured using a periodontal probe (BPWHO: Single, Osung, South Korea) to evaluate the periodontal condition. Patients exhibiting a significant amount of calculus will be referred for scaling and root planning procedures. Skilled oral and maxillofacial surgeons will carry out the interventions following standardized operating procedures. Video demonstrating the clinical and surgical procedures will be presented during the initial calibration meeting to ensure consistency across all operators.

The extraction of mandibular third molars will be carried out in a sterile environment under local anesthesia, minimizing damage to both the soft tissue (gingiva) and the hard tissue up to the crest of the bone. The surgery will adhere to the principle of minimally invasive techniques, using a split technique. To preserve the integrity of the marginal alveolar bone, an elevator will be used carefully to avoid any harm to the bone margins. Initially, a triangular access flap was created, with a releasing incision made on the tuberosity and another distal to the second molar. Then, the third molars will be split, and extraction will be performed using forceps or a dental elevator. After removing the tooth, a thorough sharp curettage will be performed to eliminate any remnants of the periodontal ligament and other soft tissues. Special attention will be given during the procedures to protect the socket walls and interproximal papillae from damage.

The fresh extraction sockets will be treated differently based on the assigned groups. In the experimental group, α-CSH material will be used to fill the socket, while no bone graft material will be applied in the control group. The amount of α-CSH used will depend on the size of the socket. Using a periosteal elevator, the material will be carefully placed and gently packed into the extraction site until it reaches the top of the socket. The graft material will be applied up to 2 mm apical to the soft tissue edge, with a maximum total dose of 3 g per patient. A membrane will be used to cover the upper side of the socket, and primary wound closure will be achieved using absorbable sutures.

Postoperatively, patients will be prescribed appropriate antibiotics and pain relievers for a duration of 7 days. They will be provided with instructions to follow a soft food diet, gargle after meals, and limit physical activities for the first 3 days following surgery. Clinical follow-up examinations will be conducted at 7 days and 14 days after the procedure. Control assessments will include panoramic radiography and CBCT scans within 3 and 6 months after the surgery to monitor the progress of bone formation.

### Adverse Event and Severe Adverse Event Assessment

Adverse events encountered during the study will be classified as serious or nonserious based on severity using the classification system established by the World Health Organization [22]. Participants who experience any adverse events will receive comprehensive medical care to address their needs. Any changes in the participant’s health status that occur between

the screening examination and the initial administration of α-CSH or associated procedures will be documented in their medical records. In the event of a severe adverse event, the principal investigator will be promptly notified within 24 hours of its occurrence. If the severe adverse event is determined to be associated with severe toxicity or infection at the transplant site, the experiment will be immediately halted to ensure participant safety.

### Sample Size

As this study focuses on feasibility rather than achieving statistical significance, formal power calculations were not conducted prior to its initiation. Instead, the emphasis lies on assessing safety, feasibility, and generating preliminary data.

With no prior data on the use of this bone graft material in humans, the sample size was determined based on practical considerations, including resource availability, feasibility of recruitment within the study timeline, and ethical concerns regarding participant risk exposure. The aim was to enroll a sample size, allowing for meaningful observations and providing valuable preliminary insights into safety and efficacy.

Furthermore, following a common rule of thumb for pilot studies, which typically suggest around 30 participants, we decided on a sample size of 30 extractions, with 15 extraction sockets in each group. The number of sockets per patient varies based on individual clinical circumstances. This sample size was deemed appropriate for assessing safety, feasibility, and generating preliminary efficacy data.

### Randomization and Blinding

The allocation of participants into the treatment and control groups will be determined through randomization. This process will be conducted using a secure computer system for central randomization. Each patient will be assigned a unique study code, and their data will be provided to the operators and research evaluators in a format that preserves patient confidentiality through coding. When evaluating the data, the radiologist and the statistician will be blinded to the treatment.

### Data Collection and Access

The study team will receive detailed guidelines and responsibilities. The medical professionals within the research team will collect the required information as outlined in Table 1. Training sessions will be conducted for the entire research team to ensure consistent data collection at each study visit. The clinical assessors and investigators will receive the data in a format that maintains patient confidentiality through coding. The participants will be monitored for a maximum duration of 6 months. The data manager will prioritize the confidentiality and security of the participant's data.

**Table 1:** Assessment of clinical trial.

	Consent form	Clinical photograph	Panoramic	CBCT^a^	Blood test	Probing	Physical examination
Preoperatively	✓	—^b^	✓	✓	✓	✓	✓
Operative day	—	✓	—	—	—	—	✓
Postop day 7	—	✓	—	—	—	—	✓
Postop day 14	—	✓	—	—	—	—	✓
Postop day 90	—	✓	✓	✓	—	✓	✓
Postop day 180	—	✓	✓	✓	—	✓	✓

^a^CBCT: cone-beam computed tomography.

^b^Not applicable.

### Outcomes

#### Feasibility Assessment

The assessment of α-CSH safety in the present context will focus on the occurrence of serious adverse events. To deem α-CSH as safe, it should not result in a higher frequency of adverse events compared to standard treatment (without socket preservation). Additionally, any adverse events that do arise should be manageable through established noninvasive procedures such as analgesics and antibiotics. The evaluation of safety will play a crucial role in determining the suitability and viability of α-CSH in the study.

Feasibility evaluation was conducted using intraoral clinical examination and photographs to assess potential complications such as inflammation, infection, dehiscence, and graft loss. Graft loss will be evaluated in the early postoperative period, within the first week, and late postoperative period. The progress of wound healing at the surgical sites will be assessed with the Landry wound healing index [23]. This index considers factors such as the color of the soft tissue (pink or red), presence of suppuration, amount of bleeding, presence of granulation tissue, and exposure of connective tissue. Based on these criteria, the healing process was categorized into 5 distinct categories, namely very poor, poor, good, very good, and excellent [23]. Patients were also asked about any pain in the surgical site or adjacent areas during the follow-up period.

#### Quantitative Bone Formation Evaluation

CBCT scans obtained before extraction, 3 months and 6 months post extraction were analyzed using Digora software (Digora for Windows 2.8.107.458 Network Client, Soredex Orion Corporation) to evaluate bone density and formation characteristics. The region of interest, corresponding to the socket preservation and grafting site, was identified based on the grafted area. The view was adjusted axially to align with the angulation of the alveolar ridge. The contours of the crestal bone were delineated, taking into account any apical recession of the alveolar crest on the buccal or lingual aspect. The buccolingual width of the alveolus was then measured 2 mm below the alveolar crest level before tooth extraction and at the follow-up time point. Changes in ridge width were calculated by subtracting the preoperative measurements from the postoperative ridge width measurements.

#### Monitoring

Continuous monitoring will be conducted by internal monitors from the Hasanuddin University Faculty of Medicine’s Ethics and Research Committee to ensure the study’s compliance with ethical guidelines. A separate Data Safety Monitoring Board will not be established as the study is associated with minimal risk. However, an annual safety report will be submitted to the Medical Research Ethics Committee of the Faculty of Medicine at Hasanuddin University.

### Statistical Analysis

Statistical analysis will be performed using SPSS 16.0 (IBM Corp). Student *t* test will be used to analyze the difference between groups and different follow-up points. The statistical significance level will be set at a *P* value of .05 or lower.

### Amendments

Any significant changes that arise during the course of the trial will be promptly reported to the ethical committee and the relevant authority. This ensures the participant’s safety and well-being, maintains the trial’s integrity, and upholds its scientific value.

### Posttrial Care

In order to ensure the safety of participants and monitor any potential long-term adverse effects of the α-CSH graft material, secondary follow-up will be conducted for all participants at 1 and 2 years. This extended follow-up period will allow for thorough evaluation and observation of any potential late-onset complications or concerns related to the use of α-CSH.

## Results

The project secured funding in 2022 and is currently in the enrollment phase, with participants being recruited and awaiting the follow-up period. The initial findings of the study are expected to be submitted for publication in late 2024.

The trial’s outcomes will be disseminated through diverse strategies, encompassing academic publications, research reports, and participation in international conferences. The results pertaining to the primary objective will be detailed in a primary manuscript designated for submission to a distinguished, high-impact, peer-reviewed journal.

## Discussion

This clinical investigation serves as the inaugural examination of human subjects to evaluate the feasibility and effectiveness of α-CSH in the regeneration of alveolar bone. Our hypothesis posits that the incorporation of α-CSH may significantly accelerate bone formation within fresh sockets, leading to a rapid restoration of bone height while mitigating the drawbacks associated with autogenous bone graft harvesting.

In dentistry, bone grafts and substitute materials are commonly used to regenerate missing hard tissue structures [24,25]. In recent years, there has been a growing interest in the use of bone graft substitutes, driven by concerns over potential immune reactions associated with allografts [1,2,18]. Challenges remain, particularly regarding graft versus host responses associated with nonautograft-derived materials [24,26]. Furthermore, one significant obstacle encountered is the limited research conducted on the safety and effectiveness of newer bone grafting materials [26,27]. Existing information about these materials is primarily derived from case reports or experimental animal models, which may raise concerns about the reliability of such data [8,26,28]. It is crucial to conduct more standardized, well-documented preclinical and clinical studies to gain a comprehensive understanding of the clinical viability and advantages of each material. This will facilitate the introduction of a wider range of commercially available products based on reliable evidence.

The α-CSH, a substance with remarkable characteristics such as high self-setting strength, excellent osteoinductive properties, biocompatibility, and angiogenesis capabilities, has emerged as a popular alternative to traditional bone grafts [29-31]. Previous in vitro and in vivo studies conducted by our team have demonstrated the ability of α-CSH to promote angiogenesis and enhance osteogenesis [14,17,29]. Given the strategic development of α-CSH as a potential biomaterial for bone regeneration, it holds promise as a unique biocomposite bone graft material to be investigated in this study [30,31]. This clinical trial represents a pioneering effort to investigate the potential regenerative capacity of α-CSH in the context of alveolar bone loss, specifically in post-extraction sockets following an odontectomy procedure.

This study aims to provide initial evidence regarding the viability of α-CSH as a bone graft material, specifically its suitability for human transplantation. The effectiveness of the intervention will be evaluated by measuring bone parameters such as bone volume, graft volume, and bone height over a 6-month period. In previous studies, histomorphometric and histological analysis has typically been used to assess the new bone formation and remineralization of the grafted sites [25,32,33]. Unfortunately, it is not feasible in the case of postodontectomy third molar due to an ethical concern for the second operation. Instead, we expect that both panoramic and CBCT imaging will provide a clear visualization of bone volume and density in the evaluated area as it has been used in previous bone regeneration studies [24,34,35]. By using radiographic techniques, we can evaluate bone production and quantify various parameters associated with bone formation [34-36]. The anticipated outcomes of this study will have significant scientific and clinical implications, enhancing our understanding of the feasibility and early efficacy of α-CSH. These findings can guide the approach to socket preservation following tooth extraction, particularly in cases involving bone augmentation for implant placement. Furthermore, this research has the potential to contribute to the development of biomaterials for clinical use, advancing the field and ultimately improving patient outcomes.

Our research encounters several limitations that warrant consideration, including the challenge of recruiting patients who meet our stringent inclusion and exclusion criteria, which poses a significant hurdle. Relying solely on hospital patients for recruitment may limit the generalizability of our findings, underscoring the need for broader sampling strategies in future studies. Additionally, the absence of previous studies to estimate sample size leaves us navigating uncharted territory, potentially impacting the precision and adequacy of our sample size determination. Despite these limitations, our study presents compelling initial data on the accelerated bone formation observed with α-CSH in fresh sockets. These findings will provide a foundation for further exploration and offer valuable insights that can inform the design and execution of larger clinical trials or studies in the future.

This protocol outlines our approach to assessing the feasibility and initial efficacy of α-CSH as a promising grafting material for alveolar bone loss, specifically in cases involving postodentectomy mandibular third molars. We aim to evaluate the potential of α-CSH as a graft material and determine its suitability in this clinical scenario.

## Abbreviations

α-CSH: α–calcium sulfate hemihydrate
CBC: complete blood count
CBCT: cone-beam computed tomography
CONSORT: Consolidated Standards of Reporting Trials
CSD: calcium sulfate dihydrate
PPD: probing pocket depth

## Data Availability

Data from this clinical trial will be available to the corresponding author upon reasonable request.
